# Effects of food waste mulch on the physicochemical quality and fungal community diversities of desert soil in Southeast Iran

**DOI:** 10.1371/journal.pone.0310518

**Published:** 2024-11-20

**Authors:** Mahdi Hajhosseini, Setareh Agha Kuchak Afshari, Mohammad Hassan Ehrampoush, Mohammad Taghi Ghaneian, Mohammad Malakoutian, Ali Asghar Ebrahimi, Hamid Reza Azimzadeh, Davood Kalantar-Neyestanaki, Mehdi Bamorovat

**Affiliations:** 1 Environmental Science and Technology Research Center, Department of Environmental Health Engineering, School of Public Health, Shahid Sadoughi University of Medical Sciences, Yazd, Iran; 2 Medical Mycology and Bacteriology Research Center, Kerman University of Medical Sciences, Kerman, Iran; 3 Environmental Health Engineering Research Center, Kerman University of Medical Sciences, Kerman, Iran; 4 Department of Medical Parasitology and Mycology, Afzalipour Faculty of Medicine, Kerman University of Medical Sciences, Kerman, Iran; 5 Department of Environmental Science, School of Natural Resources and Desert Studies, Yazd University, Yazd, Iran; 6 Department of Medical Microbiology (Bacteriology & Virology), Afzalipour School of Medicine, Kerman University of Medical Sciences, Kerman, Iran; 7 Leishmaniasis Research Center, Kerman University of Medical Sciences, Kerman, Iran; Manipal Institute of Technology, INDIA

## Abstract

In semi-arid and arid regions, mulching with various materials is one of the highly significant ways to keep soil surface coverage. This approach helps efficiently reduce drought stress and soil erosion, thus preserving soil composition and ecosystem. The research aimed to pinpoint the physicochemical alterations and fungal diversity brought on by food waste mulch (FWM) in the desert soil. An experimental field assessment was conducted from early April (spring) to late August (summer) 2021 in the soil of the Jupar desert, the main watershed of the Central Plateau, southeastern Iran. The mulch was made from a combination of clay (70%), food waste (15%), and water and sprayed in 3 plots on the Jupar desert soil surface as a case group. Moreover, 3 plots of the Jupar desert soil and clay were selected as a non-mulch-controlled surface (control groups). The physicochemical changes were studied in all groups including FWM, desert soil, and clay. Besides, the samples were cultured and checked daily to determine the growth of fungal colonies. All fungal isolates were characterized to the species level by phenotypical and molecular methods. Sequence analysis of the ITS1, 5.8S, and ITS2 regions was done. The statistical findings displayed that the physical and chemical characteristics of FWM (case group) were significantly different compared to clay and soil samples (control groups) (P<0.05). Phenotypic and genotypic analysis of the fungal consortium showed that the most frequent filamentous and yeast fungi belonged to the *Alternaria* and *Naganishia* genera, respectively. Identified fungi are classified as growth-inducing and anti-pest fungi. This study showed that adding FWM of organic matter can cause partial variety in soil fungal diversity and stabilize the desert soil due to enriching the organic matter in eroding soils.

Effects of food waste mulch on the physicochemical quality and fungal community diversities of desert soil in Southeast Iran

## Introduction

Arid and desert areas occupy 41% of the earth’s surface and 38% of the total land population lives in these areas [1]. Droughts and higher temperatures are the result of climate changes in dry and desert regions. On the other side, human activity has irreparably harmed natural resources and biodiversity via unsustainable farming techniques, agri-food systems, and unchecked urbanization [2]. The high pace of biodiversity loss will have disastrous effects on mankind and the ability to feed the planet if these variables are not managed. Half of the earth’s surface is projected to become desert and dry land by 2100 [3]. Since the global population and food consumption are increasing, food waste is also increasing rapidly so 30% of food is converted into waste due to environmental, social, and economic. Estimates indicate that 8 to 10 percent of global greenhouse gas emissions are from food not consumed [4]. In this regard, using food waste as a mulch to stabilize the soil might reduce the environmental effects of food waste.

In arid and semi-arid areas, mulching with several materials such as straw and gravel is one of the most significant ways of protecting soil surface coverage. Mulching helps effectively decrease drought stress and soil erosion, thus preserving soil construction and the ecosystem [5, 6]. Surface mulching with various materials can considerably change soil environment, and local hydrological cycles [7, 8]. Mulch can be applied to raise the average temperature of the soil [5]. Additionally, mulch may reduce vaporization and surface runoff, increase the effectiveness of water consumption, and provide a better environment for plant development [9, 10]. According to research, the best mulch layer for maintaining excellent crop yields is 7–8 cm thick [5]. However, several investigations show that different forms of mulch often behave differently in varied soil ecosystems [11–13]. The decrease in water erosion, and nutrient loss can influence the soil’s biological features and microenvironment [14]. Moreover, the composition of soil bacterial and fungal microorganisms is related to the qualities and changes in soil properties [15–17]. On the other hand, soil microbial communities are affected by soil property changes [16, 18]. The changes in soil moisture (SM) and temperature could affect soil bacterial community composition. Bacterial diversity is also influenced by variations in the organic carbon (OC) and total nitrogen (TN) of the soil [18]. The SM, pH, temperature, dissolved OC, nitrate-nitrogen, and other factors have an impact on the diversity of the soil fungus community [16]. Soil bacteria and fungi have a vital role in the functioning of terrestrial ecosystems, however, our knowledge of their reactions to climate modification lags considerably behind that of other organisms. However, in the worst environmental conditions, arid areas, and saline soils, various fungi were found, including the genus of *Cladosporium*, *Aspergillus*, *Penicillium*, *Aureobasidium*, and *Fusarium* [19]. Likewise, some yeast fungi such as *Filobasidium magnum*, *Naganishia albida*, and *Lipomyces spp*. contain extracellular polymeric materials that protect them from adverse environmental conditions and also increase the stability of soil structure [20, 21]. Plant growth-promoting fungi (PGPF) have a special attraction as bio-fertilizers due to multiple helpful effects on plants’ quality and quantity and a positive association with the environment [22]. In this regard, determining the physicochemical changes and identifying the diversity of PGPF brought on by the influence of food waste mulching on the main watershed of the Jupar desert soil, the Central Plateau in southeast Iran, was the goal of the current research.

## Material and methods

### Ethical consideration

The Research Ethics Committee of Kerman University of Medical Sciences approved the study (Ethics no: IR.KMU.REC. 1399.614).

### Site description

This experimental field assessment was conducted from early April (spring) to late August (summer) 2021 along with the western side of the Lut desert, 30 km from the city of Kerman, in the Jupar desert, located in southeastern Iran. Jupar is a part of the Daranjir desert, which is considered one of the closed watersheds of Iran. The Daranjir desert is a sub-category of the main watershed of the Central Plateau. Its geographical coordinates are 30.1632 ° N, and 57.1162 ° E. This desert area has cold, dry winters and scorching, dry summers. With a low temperature of -9 °C and a high temperature of 46 °C, the average yearly temperature is 17 °C. Between 2020 and 2021, the relative humidity fluctuated between 5 and 25 percent and the average annual rainfall ranged from 97 to 128 mm.

### Soil sampling and physicochemical properties analysis

The mulch was made from a combination of clay (700 gr) and shredded food waste up to a size of 1cm (including vegetable and fruit waste (100 gr) plus leftover food waste (25 gr) plus garden waste or straw (25 gr)), and water. The experimental study was designed based on a full factorial model (split block design). As a case group, food waste mulch (FWM) was sprayed by a Flicker hand-wall sprayer that absorbs to a depth of three to five centimeters from the soil surface. FWM was sprayed in three plots (two meters by two meters) at a distance of 20 meters from each other on the soil surface of the Jupar desert. Also, three plots of desert soil were selected as a non-mulch-controlled surface (control groups). Following six months of mulching the sand, samples were taken at ten different sites on the case and control surfaces, ranging in depth from five centimeters to twenty centimeters. In all groups, biological tests were conducted. Experiments were performed on two levels of mulched soil and without mulch.

Three samples were collected from each plot, and after sieving, the samples were homogenized through a 2 mm sieve. Each sample was divided into three parts. One part of each sample was dried in air to determine its chemical properties. The second part was used for fungal identification. Another sample was stored at 4 °C in a refrigerator to determine SM, nutrients, and microbial biomass carbon and nitrogen.

The study of chemical quality was done in three groups, including FWM (mulch), desert soil, and clay used in FWM. Soil organic carbon (OC) was determined using wet oxidation by dichromate oxidation and titration by ammonium iron sulfate. Thus, soil pH was measured using a glass electrode meter (Sartorius PB-10, China) in soil and distilled water suspension in a ratio of 1: 5. Soil total carbon (TC) and TN were measured by an elemental analyzer (VarioEL III, Elementar, Germany). Additionally, the usual technique was used to quantify the amounts of available phosphorus (AP), available potassium (AK), and available nitrogen (AN). Inductively coupled plasma mass spectrometry (ICP-MS) was used to assess the total amounts of heavy metals in soil samples using the acid digestion procedure in accordance with the EPA 3051; USEPA, 2004 guidelines.

### Molecular examination of fungal isolates

The dilutions of 10^−1^ to 10^−3^ from soil samples were cultured on Sabouraud dextrose agar (Merck, Germany) supplemented with 0.5 μg/ml Chloramphenicol (Merck, Germany), and incubated at 28°C for 7 days. All cultured samples were checked daily to determine fungal colony growth. All fungal isolates were identified using the molecular method. DNA was extracted using the Phenol-Chloroform-glass beads protocol as described elsewhere [23]. The ITS1, 5.8S, and ITS2 regions were amplified using universal fungal primer pairs ITS1 (5-TCCGTAGGTGAACCTGCGG-3) and ITS4 (5-TCCTCCGCTTATTGATATGC-3). The thermal cycling condition was as the following program: 5 min of initial denaturation at 94°C, followed by, 35 cycles of denaturation (94°C for 30 seconds), annealing (58°C for 45 seconds), and extension (72°C for 120 seconds); and a final extension step at 72°C for 8 min [24]. PCR products were electrophoresed on 1% agarose gel stained with ethidium bromide, at 80 V for 1 h, and were visualized under a UV illuminator. Then, PCR products were sequenced (Macrogen Inc., Korea), and species identification of the isolates was obtained using the online basic local alignment search tool (BLAST) system at the website of National Center for Biotechnology Information (http://www.ncbi.nlm.nih.gov). A GenBank accession number was assigned to each sequence that was obtained for this study. The sequences were analyzed phylogenetically using Molecular Evolutionary Genetics Analysis (MEGA) software version X [25]. Redundancy analysis (RDA) was used to identify microbial interactions and environmental variables.

### Statistical analyses

Statistical analyses were performed in SPSS software (v19.0) (IBM, Armonk, NY, USA). Biological statistical analysis was performed in two groups of case and control soil. Hence, statistical analysis of soil chemical elements was performed in three groups FWM, control soil, and clay. Principal component analysis (PCA) was performed to detect differences in the microbial community structure of all soil samples using Canoco5.0. Thus, the effects of physicochemical properties of soil and the relative abundance of data were analyzed using a one-way analysis of variance and the analysis of the least significant difference (LSD). The significant level of p-value of 0.05 was considered.

## Results

### Physicochemical properties of soil

The results of physical properties, including pH, moisture content (MC), and electrical conductivity (EC) in FWM, soil, and clay samples are displayed in Table 1 (S1 File and S1 Table). Physical and chemical characteristics of FWM as compared to clay and soil samples showed significant differences. FWM boosted the organic carbon and nitrogen contents of arid soil by 4.3 and 5 times, respectively. On the other hand, FWM controlled the pH at the soil’s surface. The soil pH was controlled from alkaline to 7.49 and adjusted to the organic layer.

**Table 1:** Differences in physical properties in the soil, FWM, and clay using the least significant difference (LSD).

Sample name	Latitude	Longitude	Altitude (m)	pH	MC (%)	EC(μs/cm)	C/N
**Soil**	30°16′ 32″ N	57°11′62″ E	1745	7.80±0.27	<10±1	2370±109	12±0.5
**FWM**	30°16′ 32″ N	57°11′62″ E	1745	7.49±0.34	<11±3	1840±89	13.4±0.61
**Clay**	30°16′ 32″ N	57°11′62″ E	1745	8.10±0.39	<10±1	1910±92	12.8±0.63

Altitude: altitude of the surface sea, FWM: Food waste mulch, MC: moisture content in dry soil, EC: electrical conductivity, C/N: total carbon to total nitrogen ratio. Results are the mean of three replicates ± standard deviation.

**Fig 1:**
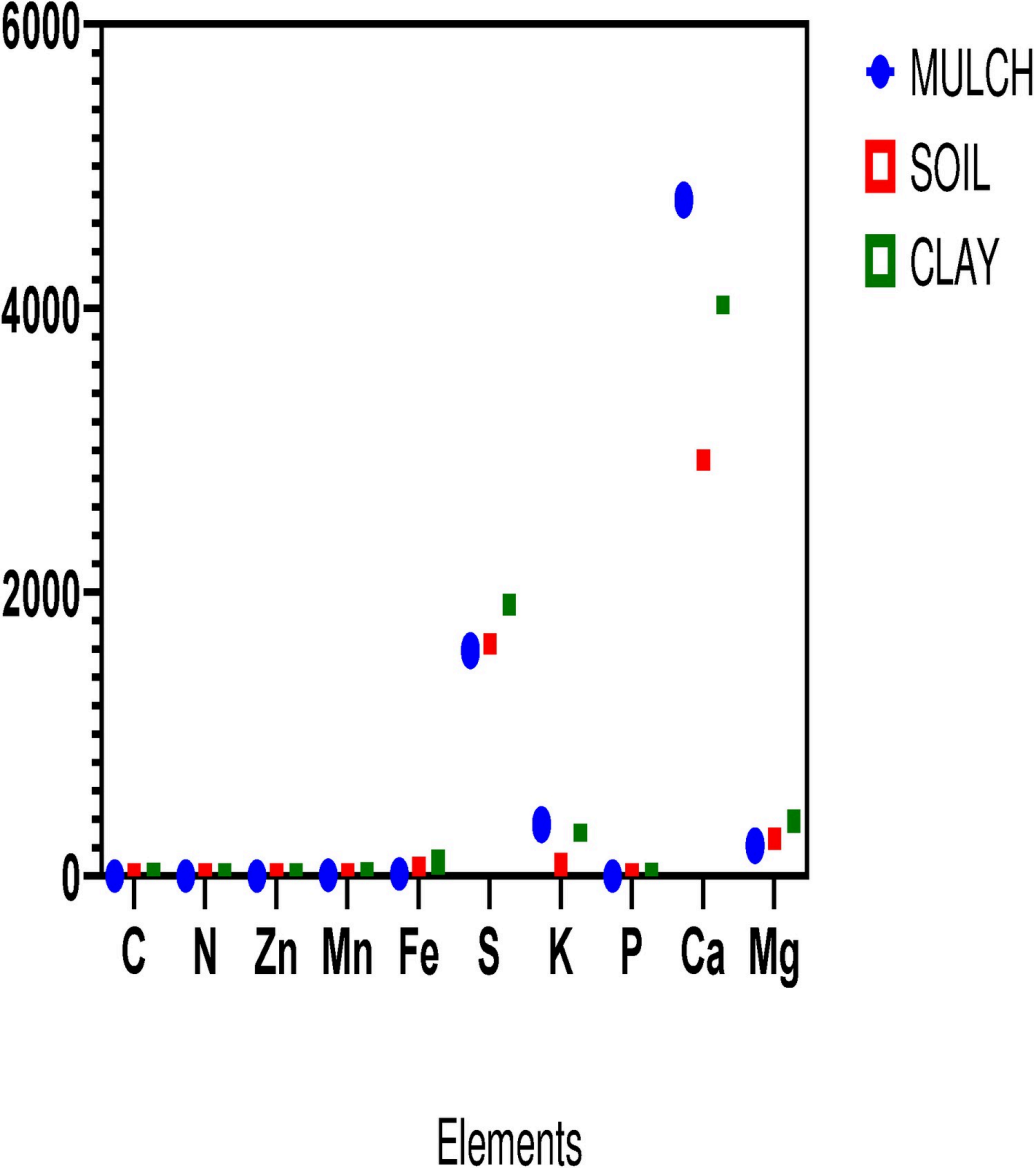
The mean of elements in FWM, soil, and clay groups.

The mean of elements in each group is presented in Fig 1. Based on the results of the least significant difference (LSD) test, there is a significant relationship between carbon and chromium changes in the desert soil and clay with FWM surface (P<0.05). Also, from the same values of all-group samples, the amount of N, K, P, Ca, Mg, and Fe elements were significant differences (P<0.05). However, the values in the Pb were not significant differences. LSD test showed that there was a significant relationship among the changes in all elements in the soil plus clay group and soil plus FWM at 0.01 level (Table 2, S2 File and S2 Table).

**Table 2:** The significant relationship between FWM plus soil (case group) and the control group (soil plus clay).

	C %	N %	K mg/kg	P mg/kg	Ca mg/kg	Mg mg/kg	Fe mg/kg	Cr mg/kg	Pb mg/kg
LSD Soil× FWM (sig)	.034^*^	.01^**^	.000^**^	.000^**^	.000^**^	.000^**^	.000^**^	.019^*^	.86
LSD Soil × Clay (sig)	.000^**^	.000^**^	.000^**^	.000^**^	.000^**^	.000^**^	.000^**^	.009^**^	.01

Least significant difference (LSD)

Mulching with a 15% food waste mixture increases the percentage of potassium up to 4 times and the percentage of calcium up to 2 times compared to the soil sample. Also, in additional analysis, there was a significant relationship between the soil plus FWM, and desert soil plus clay groups. Since the soil plus FWM group’s C/N ratio was greater than the desert soil plus clay samples, the mulched layer’s average thickness of three cm contains more organic carbon than the control soil sample and less than the clay sample. Table 3 shows organic carbon and nitrogen amounts in the FWM, soil, and clay groups (additional data and findings are found in S1 File and S1 Table).

**Table 3:** Organic carbon and nitrogen amount in the FWM, soil, and clay groups.

	C%	N%
**FWM**	0.52 + 0.048	0.053 + 0.006
**Soil**	0.1+ 0.026	0.01 + 0.001
**Clay**	4.6 + 0.4	0.3 + 0.7

**Fig 2:**
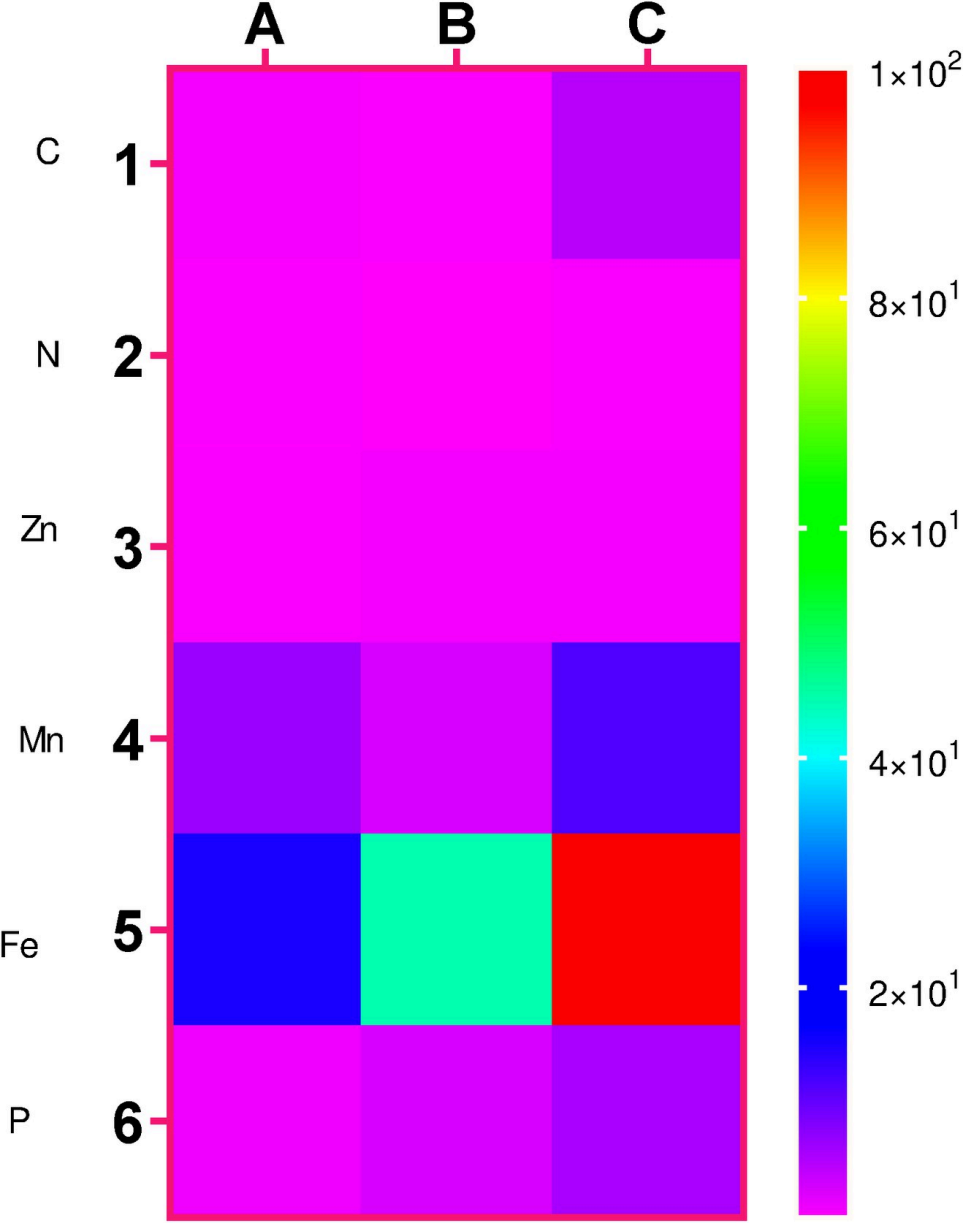
Thermal map for differences in changes of microelement in FWM (A (, soil (B), and clay (C) groups.

The thermal map of elements is shown in Fig 2.

**Fig 3:**
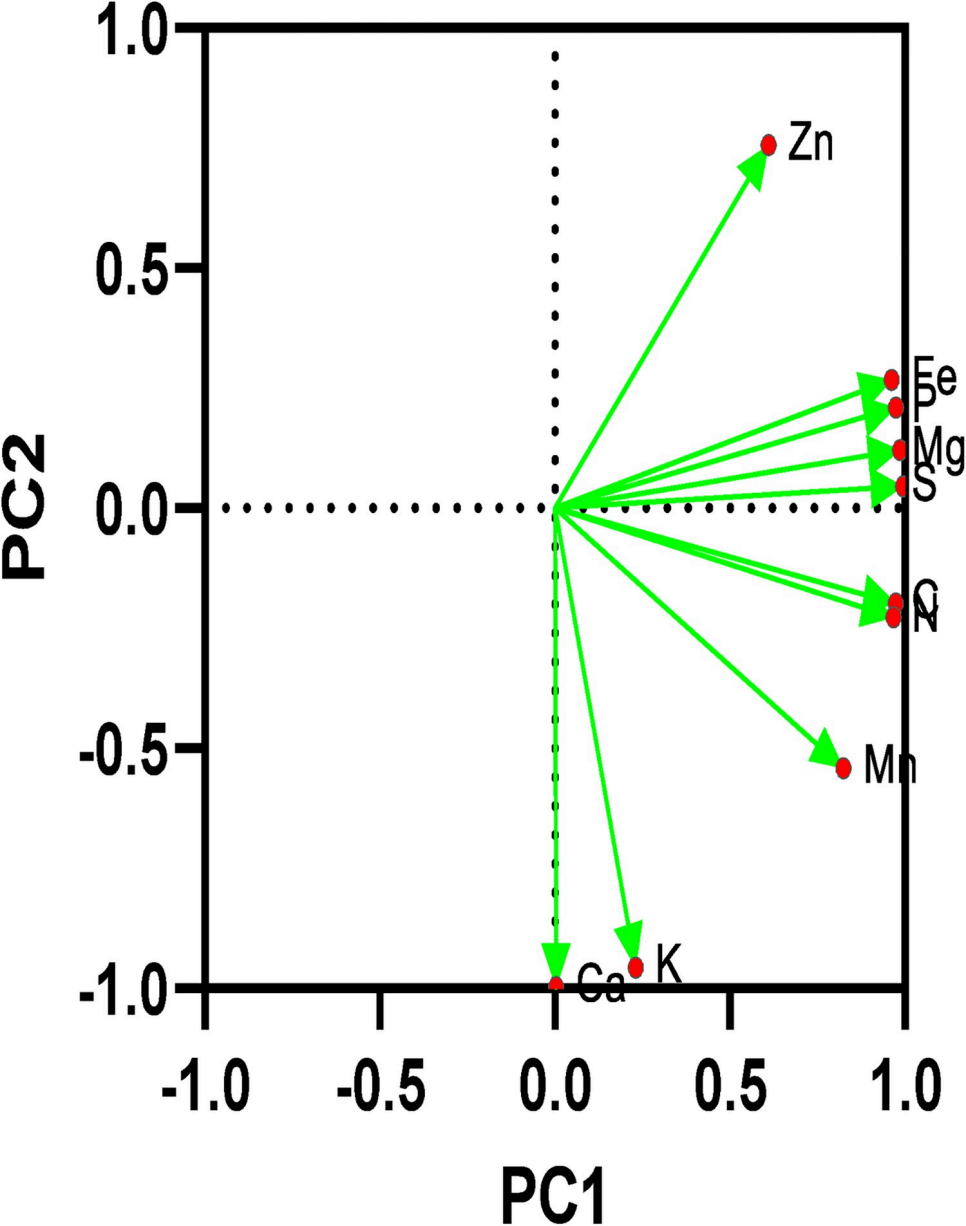
PCA between FWM, soil, and clay groups shows that 68.15% of the variances are located in PC1 and 29.96% of the variance of the group is located in PC2, which indicates that the presence and effect of Zn, C, N, Fe elements are more than Ca and K.

The results of PCA between FWM, soil, and clay groups are presented in Fig 3.

### Fungal identification

Phenotypic and genotypic analysis of fungal colonies showed that the consortium belonged to four genera and six species. Based on sequencing of the ITS rDNA region, out of eight colonies, two isolates were yeast whereas six isolates were filamentous. FWM fungi (case samples) were included *N*. *albida* (n = 1, (accession number; OP782588)), *Alternaria zantedeschiae* (n = 2, (accession numbers; OP782584 and OP782585)), *Scedosporium apiospermum* (n = 1, (accession number; OP782590)), and soil fungi (control samples) were *Cladosporium allicinum* (n = 1, (accession number; OP782589)), *N*. *adeliensis* (n = 1, (accession number; OP782587)), *A*. *alternata* (n = 1, (accession number; OP782586)), and *A*. *zantedeschiae* (n = 1, (accession number; OP782583)).

**Fig 4:**
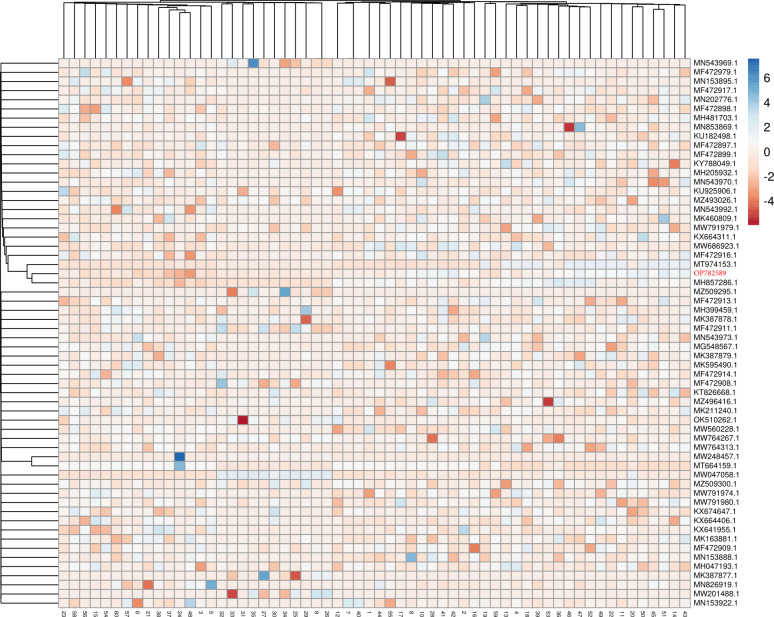
Thermal map with the corresponding dendrogram for *C*. *allicinum* (OP782589) belonging to the family of Ascomycete.

**Fig 5:**
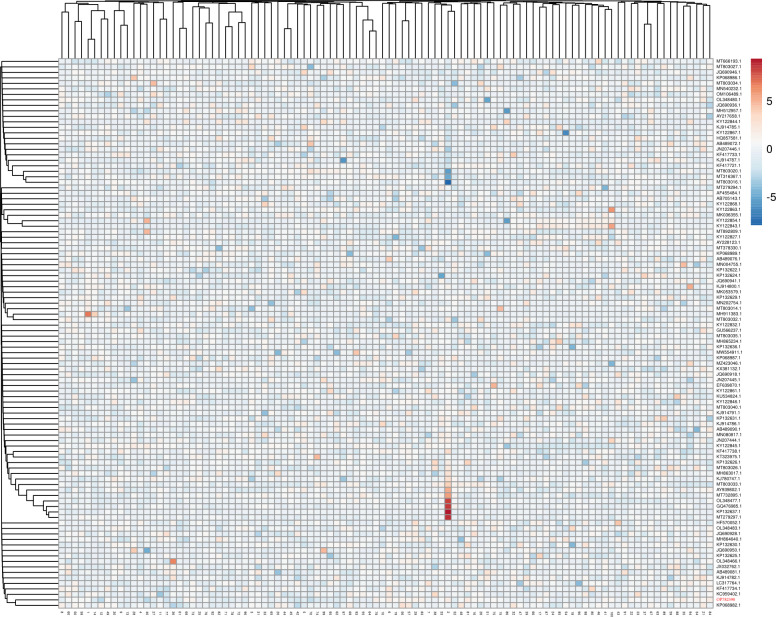
The thermal map with the corresponding dendrogram for *S*. *apiospermum* (OP782590).

Heat maps were represented, and analyzed for comparisons and understanding of where the biggest differences exist in *C*. *allicinum* isolates (Fig 4), and *S*. *apiospermum* (Fig 5).

**Fig 6:**
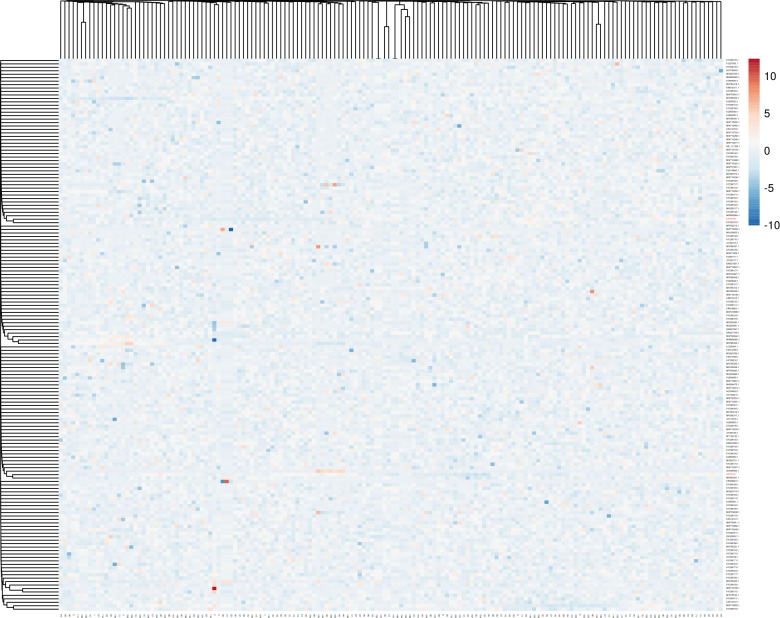
The thermal map of two *Naganishia* isolates. *N*. *albida* (OP782588) from FWM and *N*. *adeliensis* (OP782587) from soil samples.

The heat map of *Naganishia* isolates is presented in Fig 6. The basidiomycete fungus, which was found in both the FWM and desert soil sample groups, include the genus *Naganishia*. The *N*. *adeliensis* isolate from the desert soil is in Clade 3, and the highest distance is from Clade 5, while the *N*. *albida* in the FWM sample was most similar to Clade 1.

**Fig 7:**
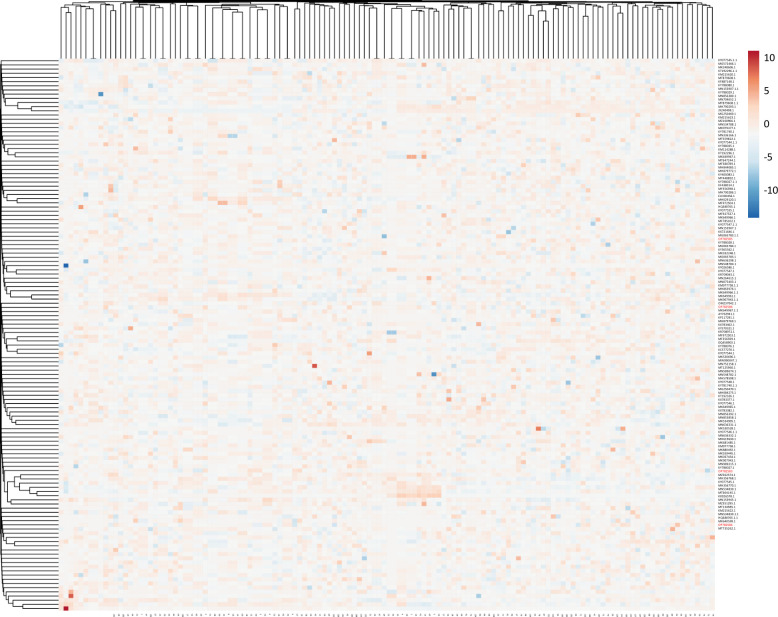
Thermal map with the corresponding dendrogram for *Alternaria* species isolated from FWM (OP782584 and OP782585) in comparison to the soil (OP782586 and OP782583) samples.

The genus *Alternaria* was the most abundant filamentous fungi in this study, but there is a genetic gap among the isolates in desert soil and FWM samples. On the other hand, the dendrogram indicates the paired genetic distances. As shown in Fig 7, two *Alternaria* strains of FWM samples are at a long genetic distance from the desert soil strains, and the desert soil isolates are genetically closer to each other (Fig 7).

## Discussion

Mulch technology is used for ecosystem changes in farmlands, forests, deserts, and urban sites [26]. There are some advantages to this procedure, including preventing evapotranspiration, buffering for soil temperature, and suppressing weed growth [27–29]. Additionally, the procedure may shield soils from wind- and water-induced compaction and erosion. Mulch may also boost plant production by maintaining soil moisture, fostering soil microbial and biological activity, and improving the soil’s physicochemical characteristics. Hence, we report the results of environmental research on the physicochemical changes and the fungal diversity in terms of adding the FWM to the Jupar desert soil in southeastern Iran. The findings showed significant changes in the physical properties of pH, OC, EC, TC, TN, and C/N in FWM (case) compared with control samples (soil and clay). Furthermore, the amount of N, K, P, Ca, Mg, and Fe elements were significant differences between the two group samples. Reports indicated mulches can create a lot of N and P in the soil, and hence reduce the need for additional nutrient inputs. The kind of mulch used might have an influence on the physical and chemical characteristics of the soil as well as crop output. Mulches made of wood chips, gravel, bark, and grass have been shown in many studies to help maintain soil moisture by lowering the rate of evaporation. Therefore, some reports have shown that organic mulches can raise the moisture content of the soil [30, 31]. Some researchers showed that the large-sized gravel particles are significant in decreasing the surface runoff, which consequently causes reduced erosion, and the loss of vital nutrients for plant growth and microorganism activity [32]. In this regard, organic mulches with their materials can decrease the space among the gravel particles. Moreover, the application of organic mulches can increase OM, N, P, K, Ca, Mg, and growth indices [33].

On the other hand, soil fertility enhancement in terms of mulching can be attributed to the promotion of microbial consortium (bacterial and fungal) activity, and consequent enhancement of the decomposition of organic material [34]. Fungi are ubiquitous microorganisms that are part of microbiota found in all natural ecosystems [35, 36]. Fungal diversity may be affected by various abiotic and biotic factors, such as temperature, pressure, ultraviolet radiation (UVR), salinity, the presence of animals, plants, and other microorganisms, sewage, and floods. Also, in habitats with inappropriate conditions, interactions among abiotic factors, such as temperature, salinity, and pH can lead to fungi applying appropriate activity for dealing with these environmental conditions [36]. Naturally, excessive salt concentration exerts significant stress on fungi and generates an adverse environment, which is thought to be associated to the restriction of fungal and microbial biodiversity. It has been shown that filamentous fungi use a variety of techniques to adapt to high salt stress when the salt content in the soil increases [37]. There is considerable evidence that many species in hot desert soil survive by their spores but without any physiological activity or cellular respiration [38].

The phenotypic and genotypic analysis of the obtained consortium showed that fungal isolates belonged to filamentous as well as yeast. In general, all samples included four genera and six species. Fungal isolates from FWM soil (case samples) included one *N*. *albida and* two *A*. *zantedeschiae*, and one *S*. *apiospermum*. The desert soil fungal isolates (control samples) included one *C*. *allicinum*, one *N*. *adeliensis*, one *A*. *alternata*, and one *A*. *zantedeschiae*. Changes in microbial communities may be associated with differences in the physical and chemical properties of soil in the study sites. Our finding showed that increasing soil nutrients due to adding FWM, does not eliminate the fungal diversity, as well this mulch strengthens the microbial structure of the soil which can result in stabilizing carbon and desert sand.

Mulch is also useful in reducing the fungus’ metabolic activity. The cellular respiration of fungus will be severely constrained due to the absence of moisture and rain for more than 300 days out of the year in the arid environment. As a result, the fungus’ ability to decompose soil organic materials will be severely constrained. Soil stabilization and greenhouse gas reduction are both possible with FWM. Two *Alternaria* species were isolated including *A*. *alternata* and *A*. *zantedeschiae*. As mentioned above, in hot and dry desert areas with low humidity and high salts, filamentous fungi, such as *Alternaria* could survive by their spores but do not have any biological activity. *Alternaria* species are well known as one of the PGPF in desert areas [22]. Studies showed the application of microbial-based consortiums like PGPF has positive effects on agriculture in terms of their multidimensional activity. In a study, *Alternaria* was isolated from roots, branches, and leaves of sage as well as the region’s soil. The previous results showed that this fungus causes a significant increase in the fresh and dry weight of the sage plant, and an increase in the content of phenolic acid, and lithospheric acids [22]. Hence, *Alternaria* species stimulate the growth of sage plant’s root and strengthen the production of its essential oil [22].

Besides, one *C*. *allicinum* and one *S*. *apiospermum* were isolated in this study. Some reports indicated *Cladosporium* species are ubiquitous endophytic fungi that are characterized as PGPF [39, 40]. Reports showed that *Cladosporium* species secreted secondary metabolites which protect plants against different biotic and abiotic stresses. On the other hand, *Cladosporium* isolates generate a broad variety of these metabolites that may act as bio-stimulating agents and have a positive impact on sustainable agriculture in addition to being abundant and inexpensive sources of nitrogen [39]. A saprophytic mold called *S*. *apiospermum* may be found in manure, polluted water, sewage, decomposing vegetation, and biological air purification systems [41]. Various factors, including organic matter content, phosphorus, and nitrogen amounts in the soil may influence the presence of *Scedosporium* species [42]. Nevertheless, further studies are needed to determine the capacity of obtained fungal isolates as PGPF.

Moreover, various soil-related yeast species were reported from different climates and soil types. There are obvious differences in the diversity and structure of the yeast community in various soils. Climate factors, such as precipitation, humidity, and intensity of solar radiation, and soil-related factors such as electrical conductivity (EC), total Phosphorus (TP), and total Potassium (TK) are the most important key factors driving the diversity of yeasts. According to the findings of our principal component analysis (PCA), OC, N, and P were associated with the frequency and diversity of yeast fungus. In this regard, it has been suggested that specific organic elements, particularly phosphorus, may be connected to the frequency of isolation of particular fungal species [43]. Besides, some basidiomycetes constantly act as natural lignocellulose destroyers by producing various extracellular enzymes that are essential for the degradation of plant biomass [44].

Two yeast species, *N*. *albida*, and *N*. *adeliensis* were isolated. *Naganishia* is a genus of fungi that comprises various yeast species distributed worldwide. *N*. *albida* can use glucose, citric acid, maltose, sucrose, trehalose, salicin, cellobiose, inositol, and many other compounds as sole carbon sources. This species can use potassium nitrate as a nitrogen source. *N*. *albida* produces urease and can stabilize the soil. It was shown that the activity of this yeast is dependent on temperature. Besides, it was demonstrated *N*. *albida* can produce extracellular polymeric substances (EPS) and survive in soils [20]. EPS are mainly polysaccharides, proteins, nucleic acids, and lipids. Biofilms are mechanically stable thanks to EPS, which also facilitates their access to nutrients and mediates their attachment to surfaces. The EPS matrix may also hold onto water, shielding the environment from drought and safeguarding bacteria. One of the roles of the EPS matrix that has been studied for decades is its capacity to agglomerate soil particles, a function that is important for the structure, health, and fertility of the soil. Because EPS has a viscous texture and ionic charges, it can act like an adhesive, binding to clay and ions and holding solid particles together [45].

The changes in environmental conditions are effective on EPS production such as temperature during the day and night, and also rainfall and dryness of the environment. Some filamentous fungi produce maximum EPS in the temperature range of 22 to 30 °C. For instance, *Alternaria* species can produce EPS, and the presence of nutrients such as iron and phosphorus are very effective in the production of EPS, which can increase the adhesion of clay and produce stable soil particles [46]. Only a few data suggest that fungus can manufacture EPS more efficiently at a temperature of 20 °C. These environmental changes have an impact on the fungal consortium that has colonized the mulch, stabilized the soil, and enhanced the cycle of nutrients available for plant development. Therefore, the presence of a fungal consortium stabilized in clay which produces the EPS in the desert environmental conditions could be useful for the sustainable production of soil grains on the sand surface of the desert. Moreover, since the EPS matrix could stabilize organic matter might be very effective to maintain the structure and reduce the respiratory activity of fungal consortium, as well as greenhouse gas production.

## Conclusion

This study showed that adding FWM with 15% of organic matter can stabilize the desert soil in terms of enriching the organic matter in eroding soils and could contribute to the partial diversity of microorganisms in the soil. Numerous physical and chemical characteristics of FWM were found to be significantly different from those of clay and soil samples. The diversity of yeast and filamentous fungi in soil, however, is only somewhat affected by FWM. However, further research with additional mulches is required to determine the characteristics and changes in soil properties, as well as the makeup of the microbial and fungi communities in the soil.

## Supporting information

S1 Table Output of analyses. (DOC)

S2 Table Output of analyses. (DOC)

S1 File Excel data file. (XLS)

S2 File Excel data file. (XLS)
