# Supplementary material for: Effects of food waste mulch on the physicochemical quality and fungal community diversities of desert soil in Southeast Iran
Source: PLoS One. 2024 Nov 20;19(11):e0310518. doi: 10.1371/journal.pone.0310518 (PMC11578483; doi:10.1371/journal.pone.0310518)
Supplement: S1 Table — (DOC) [file pone.0310518.s001.doc]

ONEWAY pH Ec CN C N BY TRE
  /STATISTICS DESCRIPTIVES HOMOGENEITY
  /MISSING ANALYSIS
  /POSTHOC=LSD ALPHA(0.05).


Oneway


Notes	
Output Created	06-APR-2024 12:00:55	
Comments		
Input	Data	C:\Users\Asus\OneDrive\Desktop\NN\sapple 1.sav	
	Active Dataset	DataSet1	
	Filter	<none>	
	Weight	<none>	
	Split File	<none>	
	N of Rows in Working Data File	27	
Missing Value Handling	Definition of Missing	User-defined missing values are treated as missing.	
	Cases Used	Statistics for each analysis are based on cases with no missing data for any variable in the analysis.	
Syntax	ONEWAY pH Ec CN C N BY TRE
  /STATISTICS DESCRIPTIVES HOMOGENEITY
  /MISSING ANALYSIS
  /POSTHOC=LSD ALPHA(0.05).	
Resources	Processor Time	00:00:00.02	
	Elapsed Time	00:00:00.02	


[DataSet1] C:\Users\Asus\OneDrive\Desktop\NN\sapple 1.sav


Descriptives	
	N	Mean	Std. Deviation	Std. Error	
					
pH	soil	9	7.80000	.268468	.089489	
	food wastemulch	9	7.49000	.337750	.112583	
	clay	9	8.10000	.389711	.129904	
	Total	27	7.79667	.410384	.078979	
Ec	soil	9	2370.0000	109.11920	36.37307	
	food wastemulch	9	1840.0000	89.20062	29.73354	
	clay	9	1910.0000	91.79869	30.59956	
	Total	27	2040.0000	257.09367	49.47770	
CN	soil	9	12.00000	.502295	.167432	
	food wastemulch	9	13.40000	.614878	.204959	
	clay	9	12.80000	.632199	.210733	
	Total	27	12.73333	.811466	.156167	
C	soil	9	.100000	.0259808	.0086603	
	food wastemulch	9	.520333	.0480651	.0160217	
	clay	9	4.600000	.3983717	.1327906	
	Total	27	1.740111	2.0801679	.4003285	
N	soil	9	.00967	.001000	.000333	
	food wastemulch	9	.05307	.005976	.001992	
	clay	9	.30000	.069282	.023094	
	Total	27	.12091	.135895	.026153	

Descriptives	
	95% Confidence Interval for Mean	Minimum	Maximum	
	Lower Bound	Upper Bound			
pH	soil	7.59364	8.00636	7.490	8.110	
	food wastemulch	7.23038	7.74962	7.100	7.880	
	clay	7.80044	8.39956	7.650	8.550	
	Total	7.63432	7.95901	7.100	8.550	
Ec	soil	2286.1236	2453.8764	2244.00	2496.00	
	food wastemulch	1771.4343	1908.5657	1737.00	1943.00	
	clay	1839.4373	1980.5627	1804.00	2016.00	
	Total	1938.2971	2141.7029	1737.00	2496.00	
CN	soil	11.61390	12.38610	11.420	12.580	
	food wastemulch	12.92736	13.87264	12.690	14.110	
	clay	12.31405	13.28595	12.070	13.530	
	Total	12.41233	13.05434	11.420	14.110	
C	soil	.080029	.119971	.0700	.1300	
	food wastemulch	.483387	.557279	.4650	.5760	
	clay	4.293784	4.906216	4.1400	5.0600	
	Total	.917224	2.562998	.0700	5.0600	
N	soil	.00890	.01044	.009	.011	
	food wastemulch	.04847	.05766	.046	.060	
	clay	.24675	.35325	.220	.380	
	Total	.06715	.17467	.009	.380	


Test of Homogeneity of Variances	
	Levene Statistic	df1	df2	
pH	Based on Mean	.525	2	24	
	Based on Median	.525	2	24	
	Based on Median and with adjusted df	.525	2	22.147	
	Based on trimmed mean	.525	2	24	
Ec	Based on Mean	.199	2	24	
	Based on Median	.199	2	24	
	Based on Median and with adjusted df	.199	2	23.191	
	Based on trimmed mean	.199	2	24	
CN	Based on Mean	.232	2	24	
	Based on Median	.232	2	24	
	Based on Median and with adjusted df	.232	2	23.170	
	Based on trimmed mean	.232	2	24	
C	Based on Mean	12.955	2	24	
	Based on Median	12.957	2	24	
	Based on Median and with adjusted df	12.957	2	8.302	
	Based on trimmed mean	12.955	2	24	
N	Based on Mean	14.343	2	24	
	Based on Median	14.408	2	24	
	Based on Median and with adjusted df	14.408	2	8.129	
	Based on trimmed mean	14.346	2	24	

Test of Homogeneity of Variances	
	Sig.	
pH	Based on Mean	.598	
	Based on Median	.598	
	Based on Median and with adjusted df	.599	
	Based on trimmed mean	.598	
Ec	Based on Mean	.821	
	Based on Median	.821	
	Based on Median and with adjusted df	.821	
	Based on trimmed mean	.821	
CN	Based on Mean	.795	
	Based on Median	.795	
	Based on Median and with adjusted df	.795	
	Based on trimmed mean	.795	
C	Based on Mean	.000	
	Based on Median	.000	
	Based on Median and with adjusted df	.003	
	Based on trimmed mean	.000	
N	Based on Mean	.000	
	Based on Median	.000	
	Based on Median and with adjusted df	.002	
	Based on trimmed mean	.000	


ANOVA	
	Sum of Squares	df	Mean Square	F	
pH	Between Groups	1.675	2	.837	7.431	
	Within Groups	2.704	24	.113		
	Total	4.379	26			
Ec	Between Groups	1492200.000	2	746100.000	79.118	
	Within Groups	226326.000	24	9430.250		
	Total	1718526.000	26			
CN	Between Groups	8.880	2	4.440	12.931	
	Within Groups	8.240	24	.343		
	Total	17.120	26			
C	Between Groups	111.211	2	55.606	1031.737	
	Within Groups	1.293	24	.054		
	Total	112.505	26			
N	Between Groups	.441	2	.221	136.909	
	Within Groups	.039	24	.002		
	Total	.480	26			

ANOVA	
	Sig.	
pH	Between Groups	.003	
	Within Groups		
	Total		
Ec	Between Groups	.000	
	Within Groups		
	Total		
CN	Between Groups	.000	
	Within Groups		
	Total		
C	Between Groups	.000	
	Within Groups		
	Total		
N	Between Groups	.000	
	Within Groups		
	Total		


Post Hoc Tests


Multiple Comparisons	
LSD  	
Dependent Variable	(I) TRE	(J) TRE	Mean Difference (I-J)	Std. Error	Sig.	
						
pH	soil	food wastemulch	.310000	.158237	.062	
		clay	-.300000	.158237	.070	
	food wastemulch	soil	-.310000	.158237	.062	
		clay	-.610000*	.158237	.001	
	clay	soil	.300000	.158237	.070	
		food wastemulch	.610000*	.158237	.001	
Ec	soil	food wastemulch	530.00000*	45.77785	.000	
		clay	460.00000*	45.77785	.000	
	food wastemulch	soil	-530.00000*	45.77785	.000	
		clay	-70.00000	45.77785	.139	
	clay	soil	-460.00000*	45.77785	.000	
		food wastemulch	70.00000	45.77785	.139	
CN	soil	food wastemulch	-1.400000*	.276225	.000	
		clay	-.800000*	.276225	.008	
	food wastemulch	soil	1.400000*	.276225	.000	
		clay	.600000*	.276225	.040	
	clay	soil	.800000*	.276225	.008	
		food wastemulch	-.600000*	.276225	.040	
C	soil	food wastemulch	-.4203333*	.1094380	.001	
		clay	-4.5000000*	.1094380	.000	
	food wastemulch	soil	.4203333*	.1094380	.001	
		clay	-4.0796667*	.1094380	.000	
	clay	soil	4.5000000*	.1094380	.000	
		food wastemulch	4.0796667*	.1094380	.000	
N	soil	food wastemulch	-.043400*	.018928	.031	
		clay	-.290333*	.018928	.000	
	food wastemulch	soil	.043400*	.018928	.031	
		clay	-.246933*	.018928	.000	
	clay	soil	.290333*	.018928	.000	
		food wastemulch	.246933*	.018928	.000	

Multiple Comparisons	
LSD  	
Dependent Variable	(I) TRE	(J) TRE	95% Confidence Interval	
			Lower Bound	Upper Bound	
pH	soil	food wastemulch	-.01658	.63658	
		clay	-.62658	.02658	
	food wastemulch	soil	-.63658	.01658	
		clay	-.93658	-.28342	
	clay	soil	-.02658	.62658	
		food wastemulch	.28342	.93658	
Ec	soil	food wastemulch	435.5192	624.4808	
		clay	365.5192	554.4808	
	food wastemulch	soil	-624.4808	-435.5192	
		clay	-164.4808	24.4808	
	clay	soil	-554.4808	-365.5192	
		food wastemulch	-24.4808	164.4808	
CN	soil	food wastemulch	-1.97010	-.82990	
		clay	-1.37010	-.22990	
	food wastemulch	soil	.82990	1.97010	
		clay	.02990	1.17010	
	clay	soil	.22990	1.37010	
		food wastemulch	-1.17010	-.02990	
C	soil	food wastemulch	-.646202	-.194464	
		clay	-4.725869	-4.274131	
	food wastemulch	soil	.194464	.646202	
		clay	-4.305536	-3.853798	
	clay	soil	4.274131	4.725869	
		food wastemulch	3.853798	4.305536	
N	soil	food wastemulch	-.08247	-.00433	
		clay	-.32940	-.25127	
	food wastemulch	soil	.00433	.08247	
		clay	-.28600	-.20787	
	clay	soil	.25127	.32940	
		food wastemulch	.20787	.28600	

*. The mean difference is significant at the 0.05 level.	
