# Supplementary material for: Effects of food waste mulch on the physicochemical quality and fungal community diversities of desert soil in Southeast Iran
Source: PLoS One. 2024 Nov 20;19(11):e0310518. doi: 10.1371/journal.pone.0310518 (PMC11578483; doi:10.1371/journal.pone.0310518)
Supplement: S2 Table — (DOC) [file pone.0310518.s002.doc]

ONEWAY Pb Cr Fe K P Ca Mg BY type
  /MISSING ANALYSIS
  /POSTHOC=LSD ALPHA(0.05).


Oneway


Notes	
Output Created	06-APR-2024 12:07:37	
Comments		
Input	Data	C:\Users\Asus\OneDrive\Desktop\NN\sapple2.sav	
	Active Dataset	DataSet1	
	Filter	<none>	
	Weight	<none>	
	Split File	<none>	
	N of Rows in Working Data File	27	
Missing Value Handling	Definition of Missing	User-defined missing values are treated as missing.	
	Cases Used	Statistics for each analysis are based on cases with no missing data for any variable in the analysis.	
Syntax	ONEWAY Pb Cr Fe K P Ca Mg BY type
  /MISSING ANALYSIS
  /POSTHOC=LSD ALPHA(0.05).	
Resources	Processor Time	00:00:00.00	
	Elapsed Time	00:00:00.02	


ANOVA	
	Sum of Squares	df	Mean Square	F	
Pb	Between Groups	.146	2	.073	46.884	
	Within Groups	.037	24	.002		
	Total	.184	26			
Cr	Between Groups	.127	2	.063	31.545	
	Within Groups	.048	24	.002		
	Total	.175	26			
Fe	Between Groups	31804.241	2	15902.120	4269.556	
	Within Groups	89.389	24	3.725		
	Total	31893.630	26			
K	Between Groups	394496.296	2	197248.148	773.241	
	Within Groups	6122.222	24	255.093		
	Total	400618.519	26			
P	Between Groups	112.727	2	56.363	664.727	
	Within Groups	2.035	24	.085		
	Total	114.762	26			
Ca	Between Groups	15287585.185	2	7643792.593	30803.343	
	Within Groups	5955.556	24	248.148		
	Total	15293540.741	26			
Mg	Between Groups	142200.000	2	71100.000	898.105	
	Within Groups	1900.000	24	79.167		
	Total	144100.000	26			

ANOVA	
	Sig.	
Pb	Between Groups	.000	
	Within Groups		
	Total		
Cr	Between Groups	.000	
	Within Groups		
	Total		
Fe	Between Groups	.000	
	Within Groups		
	Total		
K	Between Groups	.000	
	Within Groups		
	Total		
P	Between Groups	.000	
	Within Groups		
	Total		
Ca	Between Groups	.000	
	Within Groups		
	Total		
Mg	Between Groups	.000	
	Within Groups		
	Total		


Post Hoc Tests


Multiple Comparisons	
LSD  	
Dependent Variable	(I) type	(J) type	Mean Difference (I-J)	Std. Error	Sig.	95% Confidence Interval	
						Lower Bound	Upper Bound	
Pb	SOIL	FWM	.00333	.01862	.859	-.0351	.0418	
		CLAY	-.15444*	.01862	.000	-.1929	-.1160	
	FWM	SOIL	-.00333	.01862	.859	-.0418	.0351	
		CLAY	-.15778*	.01862	.000	-.1962	-.1194	
	CLAY	SOIL	.15444*	.01862	.000	.1160	.1929	
		FWM	.15778*	.01862	.000	.1194	.1962	
Cr	SOIL	FWM	-.13889*	.02114	.000	-.1825	-.0953	
		CLAY	-.15111*	.02114	.000	-.1947	-.1075	
	FWM	SOIL	.13889*	.02114	.000	.0953	.1825	
		CLAY	-.01222	.02114	.568	-.0558	.0314	
	CLAY	SOIL	.15111*	.02114	.000	.1075	.1947	
		FWM	.01222	.02114	.568	-.0314	.0558	
Fe	SOIL	FWM	-30.94444*	.90977	.000	-32.8221	-29.0668	
		CLAY	-83.16667*	.90977	.000	-85.0443	-81.2890	
	FWM	SOIL	30.94444*	.90977	.000	29.0668	32.8221	
		CLAY	-52.22222*	.90977	.000	-54.0999	-50.3446	
	CLAY	SOIL	83.16667*	.90977	.000	81.2890	85.0443	
		FWM	52.22222*	.90977	.000	50.3446	54.0999	
K	SOIL	FWM	278.88889*	7.52909	.000	263.3496	294.4282	
		CLAY	53.33333*	7.52909	.000	37.7940	68.8726	
	FWM	SOIL	-278.88889*	7.52909	.000	-294.4282	-263.3496	
		CLAY	-225.55556*	7.52909	.000	-241.0948	-210.0163	
	CLAY	SOIL	-53.33333*	7.52909	.000	-68.8726	-37.7940	
		FWM	225.55556*	7.52909	.000	210.0163	241.0948	
P	SOIL	FWM	-1.56667*	.13727	.000	-1.8500	-1.2834	
		CLAY	-4.90000*	.13727	.000	-5.1833	-4.6167	
	FWM	SOIL	1.56667*	.13727	.000	1.2834	1.8500	
		CLAY	-3.33333*	.13727	.000	-3.6166	-3.0500	
	CLAY	SOIL	4.90000*	.13727	.000	4.6167	5.1833	
		FWM	3.33333*	.13727	.000	3.0500	3.6166	
Ca	SOIL	FWM	1831.11111*	7.42590	.000	1815.7848	1846.4374	
		CLAY	733.33333*	7.42590	.000	718.0070	748.6596	
	FWM	SOIL	-1831.11111*	7.42590	.000	-1846.4374	-1815.7848	
		CLAY	-1097.77778*	7.42590	.000	-1113.1041	-1082.4515	
	CLAY	SOIL	-733.33333*	7.42590	.000	-748.6596	-718.0070	
		FWM	1097.77778*	7.42590	.000	1082.4515	1113.1041	
Mg	SOIL	FWM	-40.00000*	4.19435	.000	-48.6567	-31.3433	
		CLAY	-170.00000*	4.19435	.000	-178.6567	-161.3433	
	FWM	SOIL	40.00000*	4.19435	.000	31.3433	48.6567	
		CLAY	-130.00000*	4.19435	.000	-138.6567	-121.3433	
	CLAY	SOIL	170.00000*	4.19435	.000	161.3433	178.6567	
		FWM	130.00000*	4.19435	.000	121.3433	138.6567	

*. The mean difference is significant at the 0.05 level.	
